# Advances in the Biosynthetic Pathways and Application Potential of Plasmalogens in Medicine

**DOI:** 10.3389/fcell.2020.00765

**Published:** 2020-08-31

**Authors:** Yulong Zhou, Ning Yu, Jie Zhao, Zhenming Xie, Zhaonan Yang, Bing Tian

**Affiliations:** ^1^Key Laboratory for Green Processing of Chemical Engineering of Xinjiang Bingtuan, School of Chemistry and Chemical Engineering, Shihezi University, Shihezi, China; ^2^MOE Key Laboratory of Biosystem Homeostasis and Protection, College of Life Sciences, Zhejiang University, Hangzhou, China; ^3^Department of Applied Biological Science, Zhejiang University, Hangzhou, China

**Keywords:** plasmalogens, biosynthesis, anaerobic, oxygen-dependent, aging disease

## Abstract

Plasmalogens are a special class of polar glycerolipids containing a vinyl-ether bond and an ester bond at sn-1 and sn-2 positions of the glycerol backbone, respectively. In animals, impaired biosynthesis and regulation of plasmalogens may lead to certain neurological and metabolic diseases. Plasmalogens deficiency was proposed to be strongly associated with neurodegenerative and metabolic diseases, such as Alzheimer’s disease (AD) and Parkinson’s disease (PD), and appropriate supplement of plasmalogens could help to prevent and possibly provide therapy of these diseases. Plasmalogens evolved first in anaerobic bacteria with an anaerobic biosynthetic pathway. Later, an oxygen-dependent biosynthesis of plasmalogens appeared in animal cells. This review summarizes and updates current knowledge of anaerobic and aerobic pathways of plasmalogens biosynthesis, including the enzymes involved, steps and aspects of the regulation of these processes. Strategies for increasing the expression of plasmalogen synthetic genes using synthetic biology techniques under specific conditions are discussed. Deep understanding of plasmalogens biosynthesis will provide the bases for the use of plasmalogens and their precursors as potential therapeutic regimens for age-related degenerative and metabolic diseases.

## Introduction

**FIGURE 1:**
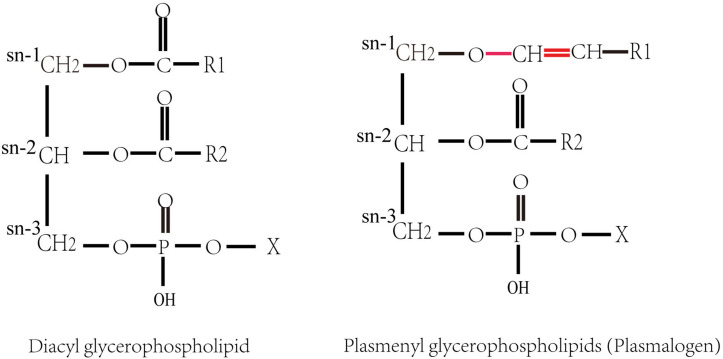
Structures of diacyl glycerophospholipid and plasmenyl glycerophospholipids (plasmalogens). X denotes the polar head group, such as ethanolamine or choline. R1 and R2 denote the hydrocarbon chains at the sn-1 and the sn-2 positions, respectively.

Plasmalogens (1-*O*-alk-1′-enyl 2-acyl glycerol phospholipids and glycolipids), also called plasmenyl phospholipid and plasmenyl glycolipids, are a special group of polar lipids, accounting for approximately 18–20 mol% of the total phospholipids in cell membranes of almost all mammalian. They are the constituents of biomembranes, which has a diversity of functions such as cell homeostasis, signaling and neural transmission (Ferlay et al., 2015; Dean and Lodhi, 2018). Plasmalogen contains a vinyl ether (-O-CH = CH-)-linked chain at sn-1 position and an ester chain at sn-2 position of glycerol backbone (Snyder, 1999; Braverman and Moser, 2012), respectively (Figure 1). Plasmalogens in animal tissues usually have a polyunsaturated acyl chain at the sn-2 position. Most of the polyunsaturated fatty acids (PUFAs) at sn-2 of plasmalogens are docosahexaenoic acid (DHA; C22:6 n-3) or arachidonic acid (AA; C20:4 n-6) in animals (Nagan and Zoeller, 2001). The representative plasmalogens in mammalian tissues are plasmalogen phosphatidylethanolamine (PlsEtn) and phosphatidylcholine plasmalogen (PlsCho) (Heymans et al., 1983), and a small portion is present as plasmenylserine (PlsSer) and phosphatidic acid plasmalogen (Deeley et al., 2009; Ivanova et al., 2010; Nagy et al., 2012). PlsEtns constitute up to 50% of ethanolamine containing glycerophospholipids in the brain (Maeba et al., 2018).

Plasmalogens evolved first in anaerobic bacteria, but they are absent in facultative and aerobic bacteria except for Myxobacteria, which were recently found to use an oxygen-dependent synthetic pathway (Lorenzen et al., 2014; Gallego-García et al., 2019). Plasmalogens are not found in fungi or plants (Goldfine, 2010). It has been proposed that plasmalogen biosynthesis requiring molecular oxygen appeared later in animal cells as respiration evolved, indicating the emergence, disappearance and recurrence of plasmalogens during evolution. This disrupted evolution of plasmalogen may be due to the sensitivity of plasmalogens to reactive oxygen species (ROS) after the concentration of oxygen increased in the early earth’s history, which will cause rapid degradation at the vinyl ether bond. However, the ability of higher organisms to use plasmalogen in an advantageous manner with special features of plasmalogens as found in animals, including antioxidant capacity, intracellular signaling and preservation of transmembrane ion gradients, may account for their reappearance (Goldfine, 2010; Lorenzen et al., 2014).

Lipid metabolism abnormalities are related to the occurrence of many human diseases (Fhaner et al., 2012). Plasmalogens are highly expressed in the nervous system and play an important role in many cellular functions of neurons (Maeba et al., 2018). Defects in plasmalogen synthesis are associated with neurodegenerative and metabolic diseases, such as Zellweger syndrome, Alzheimer’s disease (AD), and Parkinson’s disease (PD) (Yamashita et al., 2017; Dean and Lodhi, 2018). Among them, AD is an age-related progressive neurodegenerative disease and the cause of common dementia symptoms. The number of AD patients might reach more than 74 million worldwide by 2030, while the pathogeny of AD remains unclear (World Alzheimer Report, 2015). Plasmalogens were considered to be one of the oxidation targets of AD (Wood et al., 2010). The level of plasmalogens in blood and cerebrospinal fluid of AD patients is decreased (Goodenowe et al., 2007), and serum PlsEtn was suggested to be one of the cognitive decline markers (Maeba et al., 2018). In recent years, increasing studies demonstrated that supplemental of plasmalogens can be used to treat the symptoms of AD patients. Patients with mild AD showed a significant decrease in plasma PlsEtn in the placebo group than in the treatment group with oral administration of plasmalogens, and plasmalogens may improve cognitive functions of mild AD (Fujino et al., 2017). Moreover, serum plasmalogen levels have been used to diagnose and successfully stratify AD patients (Wood et al., 2015). These illustrate the importance of comprehensive understanding of the functions and biosynthesis of plasmalogens, which might be developed as a potential medicine for AD.

This review summarized the enzymes (genes) and steps involved in the aerobic and anaerobic pathways of plasmalogens biosynthesis. The significance of recently found important genes and strategies for increasing the production and application potential of plasmalogens in medicine are discussed.

## Plasmalogens Biosynthesis in Anaerobic Bacteria

The biosynthesis of plasmalogens differs in synthetic enzymes (genes) and substrates between anaerobic microorganisms and animals. In anaerobic bacteria, glycerol 3-phosphate has been confirmed as the precursor for plasmalogen synthesis (Hill and Lands, 1970; Prins and Van Golde, 1976), while dihydroxyacetone phosphate (DHAP) is the precursor of plasmalogens in animals. The enzymes related to phospholipid and plasmalogens synthesis identified in anaerobic bacteria up to date are listed in Table 1.

**TABLE 1:** Enzymes related to plasmalogens biosynthesis in anaerobic bacteria.

Enzyme name	Function descriptions
PlsX/Y	Glycerol 3-phosphate acyltransferase
CdsA	CDP-diacylglycerol synthase
PgsA	Phosphatidylglycerol phosphate synthase
PssA	Phosphatidylserine synthase
Psd	Phosphatidylserine decarboxylase
PgpA/PgpB	PGP phosphatases

*CDP, cytidine diphosphate; PGP, phosphatidylglycerol phosphate.*

By measuring the kinetics of incorporation of ^32^Pi and ^14^C into the diacylphosphatides and plasmalogens using radioautography, the reaction steps of anaerobic pathway were investigated. In *Clostridium beijerinckii* ATCC 6015, rapid incorporation of ^32^Pi into diacylphosphatidylethanolamine (diacyl-PtdEtn) and diacyl N-monomethyl PtdEtn, and a delayed incorporation into their corresponding plasmalogens, indicating that diacylphosphatide could be substrates for the corresponding plasmalogens (Baumann et al., 1965). A subsequent ^14^C-labeled acetate incorporation study also demonstrated a consistent precursor-product relationship between the chains attached to the phosphatidyl and alkyl-1-alkenyl ethers (Hagen and Goldfine, 1967). Moreover, labeling of the plasmalogen forms of phosphatidylglycerol (PtdGro) and cardiolipin is also delayed relative to the labeling of all acyl forms in *C*. *beijerinckii* (Koga and Goldfine, 1984). When hydroxylamine was added to the medium to block the decarboxylation of phosphatidylserine (PtdSer), there was initially 95% diacyl form and a 5% plasmalogen form of PtdSer; PtdSer was rapidly decarboxylated to form PtdEtn followed by the PlsEtn after the removal of hydroxylamine (Goldfine, 2017).

**FIGURE 2:**
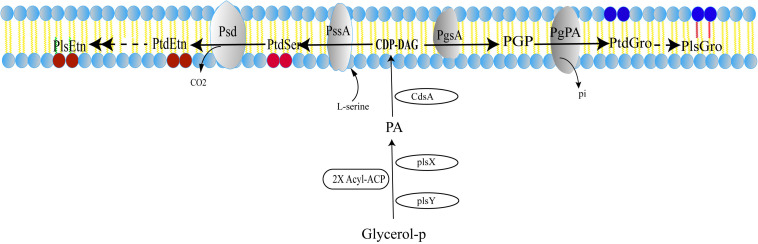
Anaerobic pathway for plasmalogen synthesis in bacteria. PA, phosphatidic acid; CDP-DAG, CDP-diacylglycerol; PGP, phosphatidylglycerol 3-phosphate; PtdEtn, phosphatidylethanolamine; PtdSer, phosphatidylserine; PtdGro, phosphatidylglycerol; PlsGro, phosphatidylglycerol plasmalogen; PlsEtn, phosphatidylethanolamine plasmalogen. The detail of enzyme and mechanism leading from the diacyl phospholipids to plasmalogens is still unknown.

A pathway of plasmalogen synthesis in anaerobic bacteria was proposed as shown in Figure 2 (Raetz and Dowhan, 1990; Dowhan, 1997; Zhang and Rock, 2008; Goldfine, 2010, 2017). First, fatty acyl-carrier protein (ACP) and glycerol 3-phosphate serve as precursors of phosphate acid (PA) under the catalysis of PlsX and PlsY. PA with cytidine triphosphate (CTP) is converted to cytidine diphosphate diacylglycerol (CDP-DAG) using CDP-diacylglycerol synthase (CdsA). Next, two additional transformations are required to produce PtdEtn or PtdGro. For PtdEtn synthesis, CDP-DAG can be converted to phosphatidylserine (PtdSer) using PtdSer synthase (PssA), and then PtdSer is converted to PtdEtn by PtdSer decarboxylase (Psd). For the branch of PtdGro synthesis, CDP-DAG is converted to phosphatidylglycerol 3-phosphate (PGP) by PGP synthase (PgsA) and then the 3-phosphate can be removed by a PGP phosphatase (PgpA or PgpB) to generate PtdGro (Dowhan, 1997). Finally, PtdEtn and PtdGro will be transformed into PlsEtn and PlsGro, respectively, under the catalysis of unknown enzymes. Although plasmalogens have been identified in anaerobic bacteria for nearly 50 years (Wegner and Foster, 1963; Goldfine, 1964), gene(s) and mechanism corresponding to the formation of the vinyl ether bond of plasmalogens remain unclear.

## The Oxygen-Dependent Pathway of Plasmalogens Biosynthesis

Phosphatidylethanolamine plasmalogen is the basic components of cell bilayers, accounting for about 20% of human phospholipids (Farooqui and Horrocks, 2001; Lessig and Fuchs, 2009; Braverman and Moser, 2012; Dorninger et al., 2015; Dean and Lodhi, 2018). Particularly, high concentrations of PlsEtn were found in the brain, retina, and other nervous tissues, accounting for 60% and 80% of the total ethanolamine phospholipids in gray and white matter, respectively (Saab et al., 2014). The plasmalogen-associated genes in animals have been studied as listed in Table 2. Among them, plasmanyl desaturase (1′-alkyl desaturase) is a predicted unstable membrane enzyme that remains to be identified for many years. In a recent report, the enzyme CarF found in an aerobic bacterium Myxobacteria was confirmed to be the 1′-alkyl desaturase for the last step of plasmalogen formation (Gallego-García et al., 2019). Its homolog TMEM189 was successively identified in mice and human (Gallego-García et al., 2019; Werner et al., 2020). Knock out of the animal homolog in human cell lines resulted in the deficiency of plasmalogens, indicating that TMEM189 is needed to catalyze the final step in plasmalogen synthesis in human cells (Gallego-García et al., 2019).

**TABLE 2:** Enzymes related to plasmalogen biosynthesis in animals.

Enzyme name	Function descriptions
FAS	Fatty acid synthase
ACS	Acyl-CoA synthase
FAR-1/2	Fatty acyl-CoA reductase 1 or 2
DHAP-AT	DHAP acyltransferase
ADHAP-S	Alkyl DHAP synthase
AADHAP-R	Alkyl/acyl-DHAP-reductase
AAG3P-AT	Alkyl/acyl-glycero-3-phosphate acyltransferase
PH	Phosphohydrolase
E-PT	Ethanolamine phosphotransferase
TMEM189 homolog	plasmanylethanolamine desaturase

*DHAP, dihydroxyacetone phosphate.*

**FIGURE 3:**
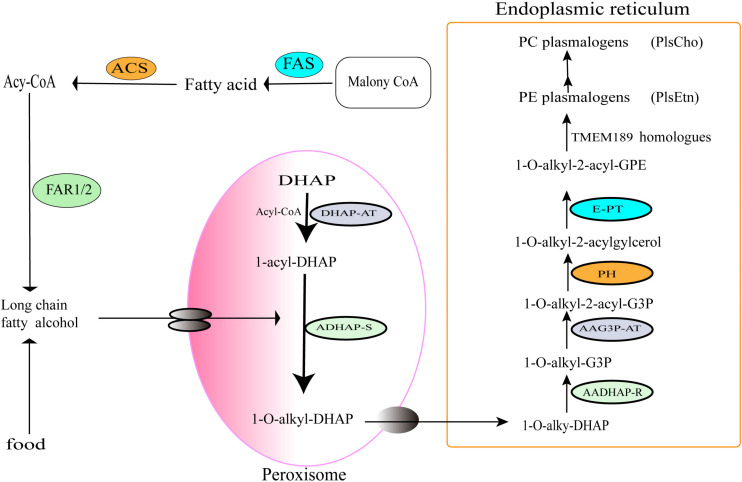
Oxygen-dependent pathway of plasmalogens biosynthesis in animals. The enzymes involved in the indicated steps: FAS, fatty acid synthase; ACS, acyl-CoA synthase; FAR1/2, fatty acyl-CoA reductase 1 or 2; DHAP-AT, DHAP acyltransferase; ADHAP-S, alkyl-DHAP synthase; AADHAP-R, NADPH:alkyl-DHAP oxidoreductase; AAG3P-AT, acyl-CoA:1-alkyl-2-lyso-sn-glycero-3-phosphate acyltransferase; PH, 1-alkyl-2-acyl-sn-glycero-3-phosphate phosphohydrolase; E-PT, CDP-ethanolamine:1-alkyl-2-acyl-sn-glycerol ethanolamine phosphotransferase; TMEM189 homologs, plasmanylethanolamine desaturases.

Plasmalogen biosynthesis occurs in peroxisomes, an oxidative organelle found in virtually all eukaryotic cells, and terminates in the endoplasmic reticulum (ER) (Wallner and Schmitz, 2011). Based on the identified enzymes in previous publications (Wallner and Schmitz, 2011; Gallego-García et al., 2019; Werner et al., 2020), an overview of the synthetic pathway is proposed in Figure 3.

Plasmalogens biosynthesis in peroxisomes of animals starts with acyl-CoA and DHAP. Under the action of DHAP-acyltransferase (DHAP-AT), DHAP is converted into 1-*O*-acyl DHAP, and then the acyl chain is replaced by a long-chain fatty alcohol from Acyl-CoA synthesis pathway (McIntyre et al., 2008) or from foods. DHAP-acyltransferase is also known as glyceronephosphate *O*-acyltransferase (GNPAT) (Nagan and Zoeller, 2001), which initiates the esterification of DHAP with a long-chain acyl-CoA (Hajra, 1997). Next, alkyl DHAP synthase (ADHAP-S) catalyzes the replacement of acyl group of 1-*O*-acyl DHAP with a long-chain fatty alcohol to generate 1-*O* alkyl-DHAP (Hajra, 1995; Hayashi and Sato, 1997; Cheng and Russell, 2004; Honsho et al., 2010; Wallner and Schmitz, 2011). The fatty alcohols are synthesized by fatty acyl-CoA reductases 1 and 2 (Far1/2) (Hajra, 1995, 1997; Nagan and Zoeller, 2001; Wallner and Schmitz, 2011) or directly taken up from diet. Notably, the Far1 is regulated by negative feedback of cellular plasmalogen levels (Honsho et al., 2010). Therefore, the formation and supply of long-chain fatty alcohols are considered to be one of the rate-limiting steps of the plasmalogen biosynthetic pathway (Paul et al., 2019). The alkyl-DHAP is then transferred from the peroxisome into the ER.

In the ER, acyl/alkyl DHAP reductase (AADHAP-R) catalyzes the reduction of 1-*O*-alkyl-DHAP to form 1-*O*-alkyl-2-hydroxyl- sn-glycerol-3-phosphate (1-*O*-alkyl-G3P) (Hajra, 1997). Then, the acyl/alkyl-G3P-acyltransferase (AAG3P-AT) catalyzes acylation with an acyl-CoA at the sn-2 position of the 1-*O*-alkyl-G3P to form 1-*O*-alkyl-2-acyl-G3P. The phosphate group of -*O*-alkyl-2-acyl-G3P is removed by phosphohydrolase (PH) to form 1-*O*-alkyl-2-acylglycerol (Wallner and Schmitz, 2011). Next, ethanolamine phosphate head group is added to 1-*O*-alkyl-2-acylglycerol by the ethanolamine phosphotransferase (E-PT) to generate plasmanylethanolamine (1-*O*-alkyl-2-acyl-GPE) (Brites et al., 2004). Finally, plasmanylethanolamine is converted into 1-*O*-alk-1′-enyl 2-acyl phosphatidylethanolamine (PlsEtn) through the action of 1′-alkyl desaturase (TMEM189 homolog) in the presence of molecular oxygen and NADPH. Because there is no plasmenylcholine desaturase found in animals, choline plasmalogens (PlsCho) might be formed only following the hydrolysis of ethanolamine plasmalogens into form 1-*O* -(1Z-alkenyl)-2-acyl-sn-glycerol, which was then modified by choline phosphotransferase and CDP-choline (Lee, 1998).

## Perspectives of Plasmalogens Biosynthesis Using Synthetic Biology Methods and Application Potentials in Medicine

Traditionally, plasmalogens are obtained using chemical synthetic method or extraction from animal tissue. However, the need for large amounts of chemicals as well as generation of potential hazardous waste during the chemical synthetic process of plasmalogens limit the applications of the chemical synthetic method. Although plasmalogens are widely found and can be prepared from marine animals or bird tissues, the amount of plasmalogens from these natural materials are very low and only account for less than 10% of the phospholipids in cell membrane of the tissues used.

Synthetic biology is a discipline that uses biological functional elements, devices and systems to carry out targeted genetic design and transformation of living organisms, to enable cells and organisms to generate specific biological functions or produce natural materials and even to synthesize “artificial life.” Using the synthetic biology techniques, artificial PUFA biosynthetic gene cluster (BGC) including a polyketide synthase-like PUFA synthetase from Myxobacteria has been introduced into yeast *Yarrowia lipolytica*, and successfully produces the highest level of DHA (16.8% of total fatty acid) among PUFA-producing *Y. lipolytica* (Gemperlein et al., 2019).

With the elucidation of plasmalogen biosynthesis genes and pathway for aerobic organisms, especially the recent identification of 1′-alkyl desaturase responsible for the conversion of plasmanylphospholipid into plasmalogens (Gallego-García et al., 2019), designing and efficient expression of plasmalogen biosynthetic modules in engineering host cells such as yeast cells become possible. The production and composition of plasmalogens is controlled by synthetic genes and certain rate-limiting steps in biosynthesis such as Far1/2 and 1-alkyl-DHAP (Paul et al., 2019). It was found that supplementation with alkyl glycerol can increase plasmalogen levels in cultured cells (Marigny et al., 2002), animals (Brites et al., 2011), and humans (Das et al., 1992). The most commonly used alkyl glycerols to increase plasmalogen levels in mammalian research are chimyl (O-16:0), batyl (O-18:0), and selachyl (O-18:1) alcohols (Brites et al., 2011; Rasmiena et al., 2015; Tham et al., 2018). Therefore, expression of plasmalogen products can be regulated under specific conditions through the genetic circuit designing and integration of gene modules composed of plasmalogen-related genes and rate-limiting elements. Standard and modularized biological elements can be used to reconstruct the metabolic network in host cells to efficiently synthesize or improve plasmalogen products that meet needs. For anaerobic biosynthesis of plasmalogen, it is necessary for us to identify the key gene(s) responsible for the formation of the vinyl ether bond of plasmalogens in anaerobic bacteria before its synthetic biological study and application.

It has been known that cultured cells and animal tissues lacking plasmalogen are more sensitive to oxidative damage than their wild-type counterparts (Zoeller et al., 1988; Reiss et al., 1997). This is due to the presence of vinyl ether bonds making plasmalogens efficient antioxidants (Broniec et al., 2011). In particular, plasmalogens can protect unsaturated membrane lipids from oxidation by singlet oxygen and participate in the removal of various ROS (Maeba et al., 2002; Skaff et al., 2008). Plasmalogen is susceptible to cleavage by ROS, yielding products that may act as second messengers (Lorenzen et al., 2014). More importantly, plasmalogen deficiency correlates with various human neurological and aging diseases, such as AD and PD (Nadeau et al., 2019; Paul et al., 2019).

Alzheimer’s disease is a complex neurodegenerative disease characterized by progressive memory loss and progressive loss of neuronal cells mainly observed in the hippocampus (Fujino et al., 2017; Jan et al., 2017). Although the gradual accumulation of β-amyloid fibers (Aβ plaque) and abnormal forms of tau (tau tangles) inside and outside neurons are considered the neuropathology of AD, the causes and mechanisms of AD have not been fully elucidated (Fujino et al., 2017; Jan et al., 2017). Accumulation of β-amyloid in AD leads to the increase of ROS levels in cells and reduces the activity of ADHAP-S, which might result in the decrease of plasmalogens (Grimm et al., 2011). Plasmalogen levels in human serum decrease with age and reductions in alkyl PtdCho and alkyl PtdEtn levels have been observed in patients with hypertension (Graessler et al., 2009). The content of plasmalogens in the brain of AD patients after death is very low (Wood et al., 2010; Braverman and Moser, 2012). Among them, the PlsEtn decreased by about 70%(Wood et al., 2010; Onodera et al., 2015). Hossain et al. (2013, 2016) found that PlsEtn inhibited the death of hippocampal neurons by increasing the phosphorylation of Akt and ERK kinases through activating the neuronal specific orphan G-protein coupled receptors (GPCRs). In their study, pan GPCR inhibitors significantly reduce the plasmalogens-induced ERK signaling in nerve cells, indicating that plasmalogens could activate GPCR-induced signaling, Plasmalogens-mediated phosphorylation of ERK was inhibited in five of the GPCRs’ knockdown cells. Overexpression of these GPCRs enhanced the plasmalogens- mediated phosphorylation of ERK and Akt, and the GPCRs-mediated cellular signaling was reduced significantly when the endogenous plasmalogens were reduced, suggesting for the first time a possible mechanism of plasmalogens-induced cell signaling in the nervous system (Hossain et al., 2016). Direct consumption of plasmalogen or related phospholipids can be used to treat dementia. For example, DHA-PC and DHA-PS can restore the content of DHA-containing PS and PlsEtn in the brain, and significantly restore the lipid homeostasis of dementia mice (SAMP8 mice), which have a phenotype that accelerates aging (Zhao et al., 2020). Oral administration of PtdEtn rich in plasmalogens (PlsEtn) from viscera of marine animals ameliorated cognitive impairment and improved the learning ability in amyloid (Aβ)-infused rats (Yamashita et al., 2017). In recent human trials, Fujino et al. (2017) reported that oral supplementing scallop-derived purified plasmalogens (1 mg/day) for 24 weeks improved memory function of patients with mild AD. Hossain et al. (2018) reported that oral ingestion of plasmalogens can attenuate the lipopolysaccharide-induced memory loss and microglial activation in mice. These findings suggest the importance of comprehensive understanding of the functions and biosynthesis of plasmalogens, which might be developed as a potential medicine for AD. Due to the increasing need of plasmalogens, it is possible to biosynthesize plasmalogens on a large scale using synthetic biological strategy.

Parkinson’s disease is a metabolic disorder and neurodegenerative disease. The pathological feature is the abnormal aggregation of SNCA/α-synuclein in the brain and the loss of dopaminergic neurons in the substantia nigra (Ho et al., 2020). The relationship between PD and plasmalogen was controversial. Although the initial study found no changes in the PlsEtn of PD patients compared with the control group (Ginsberg et al., 1995), recent studies have found that the serum concentration of PlsEtn in PD patients is reduced and low levels of plasmalogen have also been detected in frontal lobe sebaceous rafts of PD patients (Dragonas et al., 2009; Fabelo et al., 2011). Nadeau et al. (2019) reported the neuroprotective and immunomodulatory effects of plasmalogen precursors on mice with PD. They found that the supplement of DHA-containing PlsEtn precursor PPI-1011 in the intestine of mice treated with 1-methyl-4-phenyl-1,2,3,6- tetrahydropyridine (MPTP) could not only prevent MPTP-induced decrease in PlsEtn levels but also reduce macrophage infiltration in the intermuscular plexus of MPTP-treated mice (Nadeau et al., 2019). These results indicate the potential application of PlsEtn in the treatment of PD.

## Conclusion

This review summarizes the current knowledge in the field of anaerobic and aerobic biosynthetic pathways and application potential of plasmalogens in medicine. The anaerobic biosynthesis of plasmalogens differs in synthetic genes and precursors from that of oxygen-dependent biosynthesis pathway. Two different biosynthetic pathways demonstrate the significant functions and evolution of plasmalogens in organisms. The recent identification of 1′-alkyl desaturase elucidated the aerobic plasmalogen biosynthesis pathway and opened the door to the aerobic synthesis of plasmalogens using synthetic biological strategy. Further investigation on the genes responsible for the critical step in anaerobic synthesis pathway is required for the comprehensive understanding of plasmalogens evolution and functions. Because of the relevance of plasmalogens to neurological diseases, it is increasingly important to investigate the production and application of plasmalogens as potential therapeutic strategies for treating and preventing neurodegenerative and metabolic diseases.

## Author Contributions

BT and YZ conceived the review and wrote the manuscript. NY, JZ, ZX, and ZY revised the literature and helped to writing the manuscript. BT supervised the overall project and edited the manuscript. All authors had the opportunity to discuss and comment on the manuscript.

## Conflict of Interest

The authors declare that the research was conducted in the absence of any commercial or financial relationships that could be construed as a potential conflict of interest.

## References

[B1] Baumann N. A. Hagen P. O. Goldfine H. (1965). Phospholipids of *Clostridium butyricum*. studies on plasmalogen composition and biosynthesis. *J. Biol. Chem.* 240 1559–1567. 14285491

[B2] Braverman N. E. Moser A. B. (2012). Functions of plasmalogen lipids in health and disease. *Biochim. Biophys. Acta* 1822 1442–1452. 10.1016/j.bbadis.2012.05.008 22627108

[B3] Brites P. Ferreira A. S. da Silva T. F. Sousa V. F. Malheiro A. R. Duran M. (2011). Alkyl-glycerol rescues plasmalogen levels and pathology of ether-phospholipid deficient mice. *PLoS One* 6:e28539. 10.1371/journal.pone.0028539 22163031 PMC3232224

[B4] Brites P. Waterham H. R. Wanders R. J. (2004). Functions and biosynthesis of plasmalogens in health and disease. *Biochim. Biophys. Acta* 1636 219–231. 10.1016/j.bbalip.2003.12.010 15164770

[B5] Broniec A. Klosinski R. Pawlak A. Wrona-Krol M. Thompson D. Sarna T. (2011). Interactions of plasmalogens and their diacyl analogs with singlet oxygen in selected model systems. *Free Radic Biol. Med.* 50 892–898. 10.1016/j.freeradbiomed.2011.01.002 21236336 PMC3073128

[B6] Cheng J. B. Russell D. W. (2004). Mammalian wax biosynthesis I. Identifification of two fatty acyl-Coenzyme A reductases with different substrate specifificities and tissue distributions. *J. Biol. Chem.* 279 37789–37797. 10.1074/jbc.m406225200 15220348 PMC2757098

[B7] Das A. K. Holmes R. D. Wilson G. N. Hajra A. K. (1992). Dietary ether lipid incorporation into tissue plasmalogens of humans and rodents. *Lipids* 27 401–405. 10.1007/bf02536379 1630273

[B8] Dean J. M. Lodhi I. J. (2018). Structural and functional roles of ether lipids. *Protein Cell* 9 196–206. 10.1007/s13238-017-0423-5 28523433 PMC5818364

[B9] Deeley J. M. Thomas M. C. Truscott R. J. Mitchell T. W. Blanksby S. J. (2009). Identification of abundant alkyl ether glycerophospholipids in the human lens by tandem mass spectrometry techniques. *Anal. Chem.* 81 1920–1930. 10.1021/ac802395d 19186979

[B10] Dorninger F. Brodde A. Braverman N. E. Moser A. B. Just W. W. Forss-Petter S. (2015). Homeostasis of phospholipids - The level of phosphatidylethanolamine tightly adapts to changes in ethanolamine plasmalogens. *Biochim. Biophys. Acta* 1851 117–128. 10.1016/j.bbalip.2014.11.005 25463479 PMC4331674

[B11] Dowhan W. (1997). Molecular basis for membrane phospholipid diversity: why are there so many lipids? *Annu. Rev. Biochem*. 66 199–232. 10.1146/annurev.biochem.66.1.199 9242906

[B12] Dragonas C. Bertsch T. Sieber C. C. Brosche T. (2009). Plasmalogens as a marker of elevated systemic oxidative stress in Parkinson’s disease. *Clin. Chem. Lab. Med.* 47 894–897. 19575554 10.1515/CCLM.2009.205

[B13] Fabelo N. Martín V. Santpere G. Marín R. Torrent L. Ferrer I. (2011). Severe alterations in lipid composition of frontal cortex lipid rafts from Parkinson’s disease and incidental Parkinson’s disease. *Mol. Med.* 17 1107–1118. 10.2119/molmed.2011.00119 21717034 PMC3188884

[B14] Farooqui A. A. Horrocks L. A. (2001). Plasmalogens: workhorse lipids of membranes innormal and injured neurons and glia. *Neuroscientist* 7 232–245. 10.1177/107385840100700308 11499402

[B15] Ferlay J. Soerjomataram I. Dikshit R. Eser S. Mathers C. Rebelo M. (2015). Cancer incidence and mortality worldwide: sources, methods and major patterns in GLOBOCAN 2012. *Int. J. Cancer* 136 E359–E386. 25220842 10.1002/ijc.29210

[B16] Fhaner C. J. Liu S. Ji H. Simpson R. J. Reid G. E. (2012). Comprehensive lipidome profiling of isogenic primary and metastatic colon adenocarcinoma cell lines. *Anal. Chem.* 84 8917–8926. 10.1021/ac302154g 23039336 PMC3491142

[B17] Fujino T. Yamada T. Asada T. Tsuboi Y. Wakana C. Mawatari S. (2017). Efficacy and blood plasmalogen changes by oral administration of plasmalogen in patients with mild Alzheimer’s disease and mild cognitive impairment: a multicenter, randomized, double-blind, placebo-controlled trial. *eBio Med.* 17 199–205. 10.1016/j.ebiom.2017.02.012 28259590 PMC5360580

[B18] Gallego-García A. Monera-Girona A. J. Pajares-Martínez E. Bastida-Martínez E. Pérez-Castaño R. Iniesta A. A. (2019). Bacterial light response reveals an orphan desaturase for human plasmalogen synthesis. *Science* 366 128–132. 10.1126/science.aay1436 31604315

[B19] Gemperlein K. Dietrich D. Kohlstedt M. Zipf G. Bernauer H. S. Wittmann C. (2019). Polyunsaturated fatty acid production by *Yarrowia lipolytica* employing designed myxobacterial PUFA synthases. *Nat. Commun.* 10:4055. 10.1038/s41467-019-12025-8 31492836 PMC6731297

[B20] Ginsberg L. Rafique S. Xuereb J. H. Rapoport S. I. Gershfeld N. L. (1995). Disease and anatomic specificity of ethanolamine plasmalogen deficiency in Alzheimer’s disease brain. *Brain Res.* 698 223–226. 10.1016/0006-8993(95)00931-f 8581486

[B21] Goldfine H. (1964). Composition of the aldehydes of *Clostridium butyricum* plasmalogens: cyclopropane aldehydes. *J. Biol. Chem.* 239 2130–2134. 14209938

[B22] Goldfine H. (2010). The appearance, disappearance and reappearance of plasmalogens in evolution. *Prog. Lipid Res.* 49 493–498. 10.1016/j.plipres.2010.07.003 20637230

[B23] Goldfine H. (2017). The anaerobic biosynthesis of plasmalogens. *FEBS Lett.* 591 2714–2719. 10.1002/1873-3468.12714 28617934

[B24] Goodenowe D. B. Cook L. L. Liu J. Lu Y. Jayasinghe D. A. Ahiahonu P. W. (2007). Peripheral ethanolamine plasmalogen deficiency: a logical causative factor in Alzheimer’s disease and dementia. *J. Lipid Res.* 48 2485–2498. 10.1194/jlr.p700023-jlr200 17664527

[B25] Graessler J. Schwudke D. Schwarz P. E. Herzog R. Shevchenko A. Borns-tein S. R. (2009). Top-down lipidomics reveals ether lipid deficiency in blood plasma of hypertensive patients. *PLoS One* 15:e6261 10.1371/journal.pone.006261 PMC2705678 19603071

[B26] Grimm M. O. Kuchenbecker J. Rothhaar T. L. Grösgen S. Hundsdörfer B. Burg V. K. (2011). Plasmalogen synthesis is regulated via alkyl-dihydroxyacetonephosphate-synthase by amyloid precursor protein processing and is affected in Alzheimer’s disease. *J. Neurochem.* 116 916–925. 10.1111/j.1471-4159.2010.07070.x 21214572

[B27] Hagen P. O. Goldfine H. (1967). Phospholipids of *Clostridium butyricum*. 3. Further studies on the origin of the aldehyde chains of plasmalogens. *J. Biol. Chem.* 242 5700–5708. 5633398

[B28] Hajra A. K. (1995). Glycerolipid biosynthesis in peroxisomes (microbodies). *Prog. Lipid Res.* 34 343–364. 10.1016/0163-7827(95)00013-5 8685243

[B29] Hajra A. K. (1997). Dihydroxyacetone phosphate acyltransferase. *Biochim. Biophys. Acta* 1348 27–34. 9370313 10.1016/s0005-2760(97)00120-3

[B30] Hayashi H. Sato A. (1997). Fatty alcohol synthesis accompanied with chain elongation in liver peroxisomes. *Biochim. Biophys. Acta* 1346 38–44. 10.1016/s0005-2760(97)00020-9 9187301

[B31] Heymans H. S. A. Schutgens R. B. H. Tan R. van den Bosch H. Borst P. (1983). Severe plasmalogen defificiency in tissues of infants without peroxisomes (Zellweger syndrome). *Nature* 306 69–70. 10.1038/306069a0 6633659

[B32] Hill E. E. Lands W. E. (1970). Formation of acyl and alkenyl glycerol derivatives in *Clostridium butyricum*. *Biochim. Biophys. Acta* 202 209–211. 10.1016/0005-2760(70)90239-0 5417192

[B33] Ho P. W. Leung C. T. Liu H. Pang S. Y. Y. Lam C. S. C. Xian J. (2020). Age-dependent accumulation of oligomeric SNCA/α-synuclein from impaired degradation in mutant LRRK2 knockin mouse model of Parkinson disease: role for therapeutic activation of chaperone-mediated autophagy (CMA). *Autophagy* 16 347–370. 10.1080/15548627.2019.1603545 30983487 PMC6984454

[B34] Honsho M. Asaoku S. Fujiki Y. (2010). Posttranslational regulation of fatty acyl-CoA reductase 1, Far1, controls ether glycerophospholipid synthesis. *J. Biol. Chem.* 285 8537–8542. 10.1074/jbc.m109.083311 20071337 PMC2838275

[B35] Hossain M. S. Ifuku M. Take S. Kawamura J. Miake K. Katafuchi T. (2013). Plasmalogens rescue neuronal cell death through an activation of AKT and ERK survival signaling. *PLoS One* 8:e83508. 10.1371/journal.pone.0083508 24376709 PMC3869814

[B36] Hossain M. S. Mineno K. Katafuchi T. (2016). Neuronal orphan G-protein coupled receptor proteins mediate plasmalogens-induced activation of ERK and Akt signaling. *PLoS One* 11:e0150846 10.1371/journal.pone.00150846 PMC4775022 26934370

[B37] Hossain M. S. Tajima A. Kotoura S. Katafuchi T. (2018). Oral ingestion of plasmalogens can attenuate the LPS-induced memory loss and microglial activation. *Biochem. Biophys. Res. Commun.* 496 1033–1039. 10.1016/j.bbrc.2018.01.078 29337053

[B38] Ivanova P. T. Milne S. B. Brown H. A. (2010). Identification of atypical ether-linked glycerophospholipid species in macrophages by mass spectrometry. *J. Lipid Res.* 51 1581–1590. 10.1194/jlr.d003715 19965583 PMC3035522

[B39] Jan A. T. Azam M. Rahman S. Almigeiti A. M. S. Choi D. H. Lee E. J. (2017). Perspective insights into disease progression, diagnostics, and therapeutic approaches in Alzheimer’s disease: a judicious update. *Front. Aging Neurosci.* 9:356. 10.3389/fnagi.2017.00356 29163138 PMC5671974

[B40] Koga Y. Goldfine H. (1984). Biosynthesis of phospholipids in *Clostridium butyricum*: kinetics of synthesis of plasmalogens and the glycerol acetal of ethanolamine plasmalogen. *J. Bacteriol.* 159 597–604. 10.1128/jb.159.2.597-604.1984 6746573 PMC215685

[B41] Lee T. C. (1998). Biosynthesis and possible biological functions of plasmalogens. *Biochim. Biophys. Acta* 1394 129–145. 10.1016/s0005-2760(98)00107-6 9795186

[B42] Lessig J. Fuchs B. (2009). Plasmalogens in biological systems: their role in oxidative processes in biological membranes, their contribution to pathological processes and aging and plasmalogen analysis. *Curr. Med. Chem.* 16 2021–2041. 10.2174/092986709788682164 19519379

[B43] Lorenzen W. Ahrendt T. Bozhüyük K. A. Bode H. B. (2014). A multifunctional enzyme is involved in bacterial ether lipid biosynthesis. *Nat. Chem. Biol.* 10 425–427. 10.1038/nchembio.1526 24814673

[B44] Maeba R. Araki A. Fujiwara Y. (2018). Serum ethanolamine plasmalogen and urine myo-inositol as cognitive decline markers. *Adv. Clin. Chem.* 87 69–111. 10.1016/bs.acc.2018.08.001 30342713

[B45] Maeba R. Sawada Y. Shimasaki H. Takahashi I. Uetaa N. (2002). Ethanolamine plasmalogens protect cholesterol-rich liposomal membranes from oxidation caused by free radicals. *Chem. Phys. Lipids* 120 145–151. 10.1016/s0009-3084(02)00101-9 12426083

[B46] Marigny K. Pedrono F. Martin-Chouly C. A. Youmine H. Saiag B. Legrand A. B. (2002). Modulation of endothelial permeability by 1-O-alkylglycerols. *Acta Physiol. Scand.* 176 263–268. 10.1046/j.1365-201x.2002.01037.x 12444931

[B47] McIntyre T. M. Snyder F. Marathe G. K. (2008). “Ether-linked lipids and their bioactive species,” in *Biochemistry of Lipids, Lipoproteins and Membranes*, eds Vance D. E. Vance J. E. (Amsterdam: Elsevier), 245–276. 10.1016/b978-044453219-0.50011-8

[B48] Nadeau J. Smith T. Lamontagne-Proulx J. Bourque M. Al Sweidi S. Jayasinghe D. (2019). Neuroprotection and immunomodulation in the gut of parkinsonian mice with a plasmalogen precursor. *Brain Res.* 1725:146460. 10.1016/j.brainres.2019.146460 31525350

[B49] Nagan N. Zoeller R. A. (2001). Plasmalogens: biosynthesis and functions. *Prog. Lipid Res.* 40 199–229. 10.1016/s0163-7827(01)00003-0 11275267

[B50] Nagy K. Brahmbhatt V. V. Berdeaux O. Bretillon L. Destaillats F. Acar N. (2012). Comparative study of serine-plasmalogens in human retina and optic nerve: identification of atypical species with odd carbon chains. *J. Lipid Res.* 53 776–783. 10.1194/jlr.d022962 22266369 PMC3307654

[B51] Onodera T. Futai E. Kan E. Abe N. Uchida T. Kamio Y. (2015). Phosphatidylethanolamine plasmalogen enhances the inhibiting effect of phosphatidylethanolamine on γ-secretase activity. *J. Biochem.* 157 301–309. 10.1093/jb/mvu074 25409699

[B52] Paul S. Lancaster G. I. Meikle P. J. (2019). Plasmalogens: a potential therapeutic target for neurodegenerative and cardiometabolic disease. *Prog. Lipid Res.* 74 186–195. 10.1016/j.plipres.2019.04.003 30974122

[B53] Prins R. A. Van Golde L. M. (1976). Entrance of glycerol into plasmalogens of somestrictly anaerobic bacteria and protozoa. *FEBS Lett.* 63 107–111. 10.1016/0014-5793(76)80204-9 1261671

[B54] Raetz C. R. Dowhan W. (1990). Biosynthesis and function of phospholipids in *Escherichia coli*. *J. Biol. Chem.* 265 1235–1238. 2404013

[B55] Rasmiena A. A. Barlow C. K. Stefanovic N. Huynh K. Tan R. Sharma A. (2015). Plasmalogen modulation attenuates atherosclerosis in ApoE-and ApoE/GPx1-deficient mice. *Atherosclerosis* 243 598–608. 10.1016/j.atherosclerosis.2015.10.096 26545014

[B56] Reiss D. Beyer K. Engelmann B. (1997). Delayed oxidative degra dation of polyunsaturated diacyl phospholipids in the presence of plasmalogen phospholipids in vitro. *Biochem. J.* 323 807–814. 10.1042/bj3230807 9169616 PMC1218386

[B57] Saab S. Mazzocco J. Creuzot-Garcher C. P. Bron A. M. Bretillon L. Acar N. (2014). Plasmalogens in the retina: from occurrence in retinal cell membranes to potential involvement in pathophysiology of retinal diseases. *Biochimie* 107(Pt A), 58–65. 10.1016/j.biochi.2014.07.023 25127660

[B58] Skaff O. Pattison D. I. Davies M. J. (2008). The vinyl ether linkages of plasmalogens are favored targets for myeloperoxidase-derived oxidants: a kinetic study. *Biochemistry* 47 8237–8245. 10.1021/bi800786q 18605737

[B59] Snyder F. (1999). The ether lipid trail: a historical perspective. *Biochim. Biophys. Acta* 1436 265–278. 9989259 10.1016/s0005-2760(98)00172-6

[B60] Tham Y. K. Huynh K. Mellett N. A. Henstridge D. C. Kiriazis H. Ooi J. Y. Y. (2018). Distinct lipidomic profiles in models of physiological and pathological cardiac remodeling, and potential therapeutic strategies. *Biochim. Biophys. Acta Mol. Cell Biol. Lipids* 1863 219–234. 10.1016/j.bbalip.2017.12.003 29217479

[B61] Wallner S. Schmitz G. (2011). Plasmalogens the neglected regulatory and scavenging lipid species. *Chem. Phys. Lipids* 164 573–589. 10.1016/j.chemphyslip.2011.06.008 21723266

[B62] Wegner G. H. Foster E. M. (1963). Incorporation of isobutyrate and valerate into cellular plasmalogen by *Bacteroides succinogenes*. *J. Bacteriol.* 85 53–61. 10.1128/jb.85.1.53-61.1963 13999496 PMC278089

[B63] Werner E. R. Keller M. A. Sailer S. Lackner K. Koch J. Hermann M. (2020). The TMEM189 gene encodes plasmanylethanolamine desaturase which introduces the characteristic vinyl ether double bond into plasmalogens. *Proc. Natl. Acad. Sci. U.S.A.* 117 7792–7798. 10.1073/pnas.1917461117 32209662 PMC7149458

[B64] Wood P. L. Locke V. A. Herling P. Passaro A. Vigna G. B. Volpato S. (2015). Targeted lipidomics distinguishes patient subgroups in mild cognitive impairment (MCI) and late onset Alzheimer’s disease (LOAD). *BBA Clin.* 5 25–28. 10.1016/j.bbacli.2015.11.004 27051586 PMC4802395

[B65] Wood P. L. Mankidy R. Ritchie S. Heath D. Wood J. A. Flax J. (2010). Circulating plasmalogen levels and Alzheimer Disease Assessment Scale-Cognitive scores in Alzheimer patients. *J. Psychiatry Neurosci.* 35 59–62. 10.1503/jpn.090059 20040248 PMC2799506

[B66] World Alzheimer Report (2015). *The Global Impact of Dementia: An Analysis of Prevalence, Incidence, Cost and Trends.* London: Alzheimer’s Disease International.

[B67] Yamashita S. Hashimoto M. Haque A. M. Nakagawa K. Kinoshita M. Shido Q. (2017). Oral administration of ethanolamine glycerophospholipid containing a high level of plasmalogen improves memory impairment in myloid β-infused rats. *Lipids* 52 575–585. 10.1007/s11745-017-4260-3 28551706

[B68] Zhang Y. M. Rock C. O. (2008). Membrane lipid homeostasis in bacteria. *Nat. Rev. Microbiol.* 6 222–233. 10.1038/nrmicro1839 18264115

[B69] Zhao Y. C. Zhou M. M. Zhang L. Y. Cong P. X. Xu J. Xue C. H. (2020). Recovery of brain DHA-containing phosphatidylserine and ethanolamine plasmalogen after dietary DHA-enriched phosphatidylcholine and phosphatidylserine in SAMP8 mice fed with high-fat diet. *Lipids Health Dis.* 19:104. 10.1186/s12944-020-01253-3 PMC7249346 32450867

[B70] Zoeller R. A. Morand O. H. Raetz C. R. (1988). A possible role for plasmalogens in protecting animal cells against photosensitized killing. *J. Biol. Chem.* 263 11590–11596. 3403547

